# A Gene Transfer-Positive Cell Sorting System Utilizing Membrane-Anchoring Affinity Tag

**DOI:** 10.3389/fbioe.2022.930966

**Published:** 2022-06-16

**Authors:** Lele Yang, Lifang Cui, Shumin Ma, Qingqing Zuo, Qilai Huang

**Affiliations:** Shandong Provincial Key Laboratory of Animal Cell and Developmental Biology, School of Life Science, Shandong University, Qingdao, China

**Keywords:** gene delivery, affinity cell sorting, gene function study, twin-strep-tag (TST), co-transfection

## Abstract

Gene delivery efficiency is an essential limit factor in gene study and gene therapy, especially for cells that are hard for gene transfer. Here we develop an affinity cell sorting system that allows efficient enrichment of gene transfer-positive cells. The system expresses an enhanced green fluorescent protein (EGFP) fused with an N-terminal high-affinity Twin-Strep-Tag (TST) that will be anchored to the cell membrane at the out-surface through a glycosylphosphatidylinositol (GPI) membrane-anchoring structure. The EGFP permits microscopy and flow cytometry analysis of the gene transfer-positive cells, and the TST tag at the N terminal of EGFP allows efficient affinity sorting of the positive cells using Strep-Tactin magnetic beads. The cell sorting system enables efficient isolation of gene transfer-positive cells in a simple, convenient, and fast manner. Cell sorting on transfected K-562 cells resulted in a final positive cell percentage of up to 95.0% with a positive cell enrichment fold of 5.8 times. The applications in gene overexpression experiments could dramatically increase the gene overexpression fold from 10 times to 58 times, and in shRNA gene knockdown experiments, cell sorting increased the gene knockdown efficiency from 12% to 53%. In addition, cell sorting in CRISPR/Cas9 genome editing experiments allowed more significant gene modification, with an editing percentage increasing from 20% to 79%. The gene transfer-positive cell sorting system holds great potential for all gene transfer studies, especially on those hard-to-transfect cells.

## Introduction

*In vitro* gene delivery has achieved great progress (Zhao et al., 2012). Both virus-mediated and non-virus-mediated gene delivery can attain high transfection efficiency for most cells (Lundstrom, 2003; Woods et al., 2003; Heller et al., 2005; Abbasalipour et al., 2019). However, the transfection efficiency is still inadequate for the hard-to-transfect cells such as lymphoma/leukemia cells and primary cells (Huang et al., 1998; Migliaccio et al., 2000; Guven et al., 2005). Improving the positive cell percentage for these cells remains a key issue in gene function study.

Enriching gene transfer-positive cells through cell sorting is an effective strategy to increase the proportion of positive cells, especially for the hard-to-transfect cells. Existing cell sorting methods mainly include antibiotic drug screening, fluorescence-activated cell sorting (FACS), and magnetic cell sorting (MACS) (Tomlinson et al., 2013; Shields et al., 2015). The drug screening method based on drug-resistant genes has been widely used in cell biology and gene function studies (Perriere et al., 2005; Hotta et al., 2009; Moriarity et al., 2014). However, it is not applicable to suspension cells because of the incompetence in removing the dead cells killed by the drug. In practice, pre-experiment is usually obligatory to determine the drug concentration for each cell line because of their diverse sensitivity to drug treatment. In addition, it is worth noticing that the drug administration may cause toxicity and lead to unpredictable side effects on gene expression and cell signaling.

The FACS method employs vectors expressing fluorescent proteins, such as EGFP, mCherry, RFP, YFP, and BFP, and sorts the fluorescence-positive cells on a flow cytometer after gene delivery (Sutermaster and Darling, 2019; Pan and Wan, 2020). This method requires a flow cytometer equipped with a sorting module, which is expensive and not readily accessible to major labs. In addition, even though having a simple procedure, the limit in sorting speed makes it less applicable in experiments desiring a large number of cells (Sutermaster and Darling, 2019; Pan and Wan, 2020).

The common MACS method utilizes an antibody-conjugated magnetic microsphere to bind and sort the target cells expressing the corresponding antigen on the cell surface (Pan and Wan, 2020). For this purpose, H-2K^k^ (Wei et al., 2001) and truncated LNGFR (Matheson et al., 2014) are usually encoded on the vectors and will locate to the cell surface when expressed in the transfected cells. The transfection-positive cells can then be isolated using magnetic beads coupled with the corresponding antibody or binding ligand. Because these molecules themselves have important biological functions, the overexpression and membrane anchoring on the cell surface might alter the gene expression profile and the cell phenotypes. For example, LNGFR is a type I transmembrane cell surface glycoprotein of the tumor necrosis factor receptor superfamily (Dechant and Barde, 2002). Overexpression of LNGFR can promote the osteogenic differentiation of rat extraembryonic mesenchymal stem cells. Using LNGFR as the sorting marker will probably alter the normal cell signaling and produce potential influences on the experimental results (Li et al., 2017). Therefore, we still lack a fast, simple, and widely applicable system to enrich gene transfer-positive cells.

Here, we describe a versatile gene transfer-positive cell sorting system based on an affinity fluorescent tag protein encoded on a vector and will be located to the cell surface once expressed. Specifically, the tag comprises the Enhanced Green Fluorescent Protein (EGFP) with an N-terminal Twin-Strep-Tag (TST) (Schmidt et al., 2013; Maertens et al., 2015; Yeliseev et al., 2017) and a C-terminal membrane positioning signal module. The gene transfer-positive cells will express and display the affinity fluorescent tags on the cell surface and, therefore, can be sorted using Magrose Strep-Tactin magnetic beads that can bind TST tags with high affinity. This positive cell sorting system is efficient, simple, low-cost, and convenient to operate, and thus has great potential in diverse gene function research and related applications, including gene overexpression, gene knockdown, reporter gene assay, genome editing, et al.

## Results

### Design the Affinity Fluorescent Sorting Tag Protein

**FIGURE 1:**
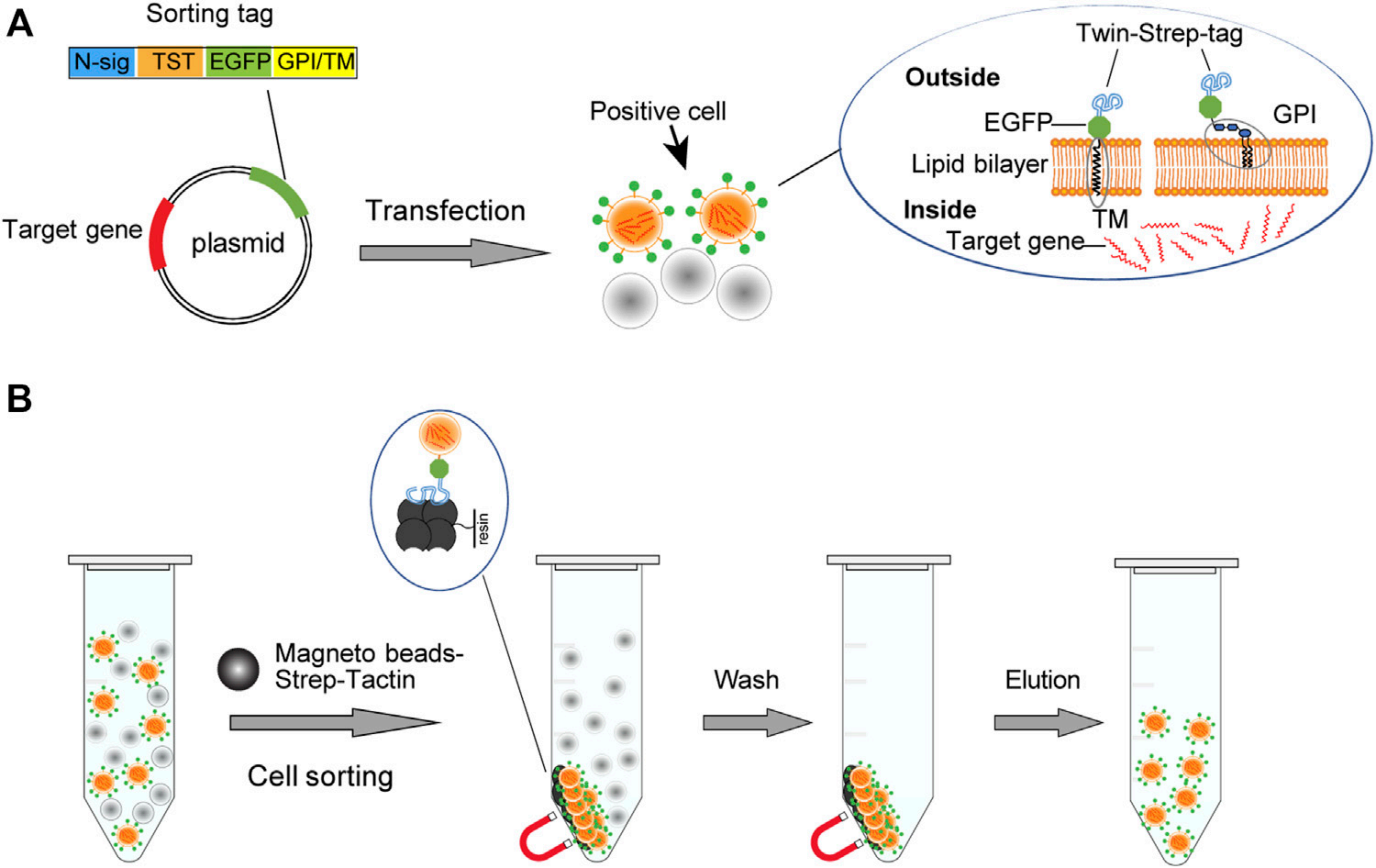
Schematic illustration of the cell sorting strategy. **(A)**The vector expresses the TST-EGFP sorting tag that targets the cell membrane through a glycosylated phosphatidylinositol (GPI) module or a transmembrane domain (TM). **(B) **The high affinity between TST and Strep-Tactin allows the gene transfection-positive cells displaying the sorting tag to be bound and enriched with the magnetic Strep-Tactin beads. Transfection-positive cells are displayed in orange. The separation of the bead/cell complex can be performed by staying on a magnetic stand or by free settling.

We design the sorting tag protein on the basis of enhanced green fluorescent protein (EGFP) to allow the identification of the gene transfer-positive cells with microscopy and flow cytometry. To realize affinity sorting of the positive cells, we fuse EGFP with an N-terminal TST tag, the dimer version of Strep-TagII that binds Strep-Tactin with high affinity (Rai et al., 2014), resembling streptavidin-biotin (Schmidt and Skerra, 2015). Further, to locate the sorting tag protein on the cell surface, we start from six membrane-anchoring modules. Among them, three membrane-anchoring motifs from BY55, DAF, and CEAM7 can anchor the protein to the outer layer of the lipid bilayer through glycosylphosphatidylinositol (GPI) molecule (Ferguson and Williams, 1988; Kinoshita et al., 2008; Paulick and Bertozzi, 2008) (Figure 1A). Three modules from ITB3, ITA5, and ITAV belong to the transmembrane domain (TMD), which can insert the eukaryotic cell membrane and anchor the molecules to the cell membrane (Ling et al., 1999; Winnard et al., 2007; Fu et al., 2019) (Figure 1A). We used SignalP-5.0 Server (Almagro Armenteros et al., 2019) to predict the corresponding module sequences and splicing sites of the given signal peptide (Supplementary Table S1). Meanwhile, to avoid evoking potential integrin signaling, we replaced all the intracellular amino acid residues involved in functional interactions (Arnaout et al., 2007) (Supplementary Table S2).

The GPI modification motif coding sequences, the transmembrane domain coding sequences, and the TST coding sequence were synthesized and joined with the reading frame of EGFP by multiplex PCR and then cloned into the pEGFP-C2 vector. Transfected cells harboring these plasmids will display corresponding sorting tags on the cell surface. Strep-Tactin magnetic beads can thus bind and enrich the transfection-positive cells (Figure 1B).

### Sorting Tags Locate to the Cell Surface

**FIGURE 2:**
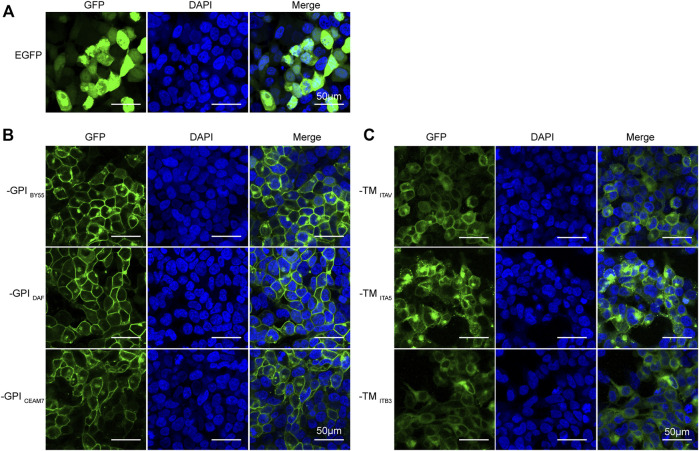
Efficient membrane-anchoring of six sorting tag variants. Laser scanning confocal microscopy of Lenti-X 293T cells transfected with the pEGFP-C2 plasmid **(A)**, three GPI-type TST-EGFP sorting tag expression plasmids **(B)**, and three TM-type TST-EGFP sorting tag expression plasmids **(C)**. DAPI stains cell nuclei. Cells were observed at ×200 magnification, scale bar = 50 μm.

First, to evaluate the cell membrane targeting ability of the six sorting tags, we transfected the corresponding expression plasmids into the Lenti-X 293T cells growing on the glass slide and observed with a confocal laser scanning fluorescence microscopy. We found that all the six sorting tags were expressed at a high level and effectively located to the cell membrane (Figure 2). The EGFP protein without membrane positioning signal was distributed throughout the whole cell (Figure 2A), and the three GPI sorting fluorescent tags exhibited an obvious membrane targeting effect (Figure 2B), at a higher degree than the three TMD sorting tags (Figure 2C). Notably, the cells expressing GPI sorting tags displayed unaffected cell morphology, but the cells expressing TMD sorting tags present a rounded shape (Figure 2C). It indicates that the integrin TMD protein overexpression might disturb the cell adhering.

### Affinity Cell Sorting Enriches Transfection-Positive Cells

**FIGURE 3:**
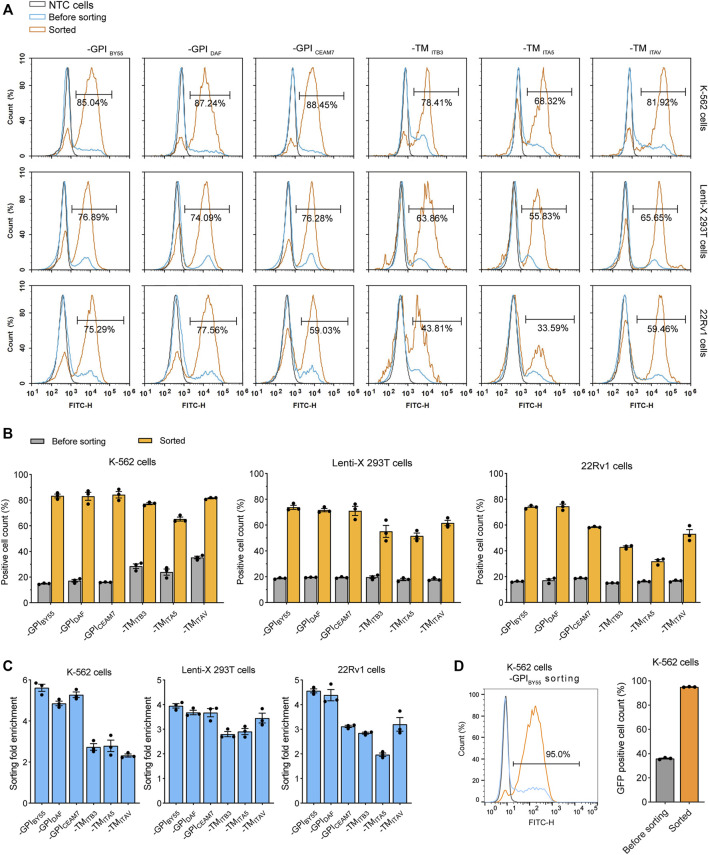
Flow cytometry analysis of cells from affinity cell sorting. **(A)** Flow cytometry histograms of K-562, Lenti-X 293T, and 22Rv1 cells transfected with six sorting tag plasmids, enriched or not by cell sorting. The histogram is presented as the normalized percentage value of the maximum value in the layer histogram. The gray layer represents the negative control cells (NTC) without transfection, and the blue layer represents the transfected cells, the orange layer represents the cells enriched by cell sorting. The percentage of fluorescence-positive cells in the enriched cells is represented. **(B)** Bar chart showing the percentage of fluorescence positive cells in the sorted K-562, Lenti-X 293T, and 22Rv1 cells determined by flow cytometry analysis. Values are from three independent biological replicates. **(C)** The enrichment fold of positive cells after affinity cell sorting with six sorting tags in K-562, Lenti-X 293T, and 22Rv1. Data from three independent biological replicates. **(D)** Flow cytometry analysis of K-562 cells transfected with TST-EGFP-GPI_BY55_ sorting tag plasmids and sorted through free settling strategy. The grey, blue, and orange layers represent negative control cells, sorting tag-transfected cells, and enriched cells. The bar chart represents data from three biological replicates.

We transfected the sorting tag vectors into three cell lines and performed affinity cell sorting with the Strep-Tactin magnetic beads to enrich the transfection-positive cells. We found that, in K-562, a suspension leukemia cell, all six sorting tags significantly enriched fluorescence positive cells. For the three GPI type sorting tags, TST-EGFP-GPI_BY55_, TST-EGFP-GPI_DAF_, and TST-EGFP-GPI_CEAM7_, the affinity sorting increased positive cell percentage from 15%, 16%, and 16%–86%, 87%, and 88%, respectively, as determined with flow cytometry (Figures 3A,B). Meanwhile, for the three TMD type sorting tags, TST-EGFP-TM_ITB3_, TST-EGFP-TM_ITA5_, TST-EGFP-TM_ITAV_, the positive cells ratio increased from 28%, 24%, and 35%–78%, 68%, and 82% respectively (Figures 3A,B). Further affinity cell sorting with the three GPI type sorting tags in the Jurkat T-cell-derived leukemia cells showed that TST-EGFP-GPI_BY55_, TST-EGFP-GPI_DAF_, and TST-EGFP-GPI_CEAM7_ increased the positive cell percentage from 13% to 67%, 77%, and 63%, respectively (Supplementary Figures S1A,B).

In the Lenti-X 293T cells, affinity cell sorting also dramatically increased the positive cell ratio from 19% to 77%, 74%, and 76%, respectively for TST-EGFP-GPI_BY55_, TST-EGFP-GPI_DAF_, and TST-EGFP-GPI_CEAM7_, and increased from 18% to 64%, 56%, and 66%, respectively for TST-EGFP-TM_ITB3_, TST-EGFP-TM_ITA5_, and TST-EGFP-TM_ITAV_ (Figures 3A,B). In addition, cell sorting on the 22Rv1 prostate cancer cell line also showed efficient enrichment of the transfection-positive cells. The three GPI type sorting tags, TST-EGFP-GPI_BY55_, TST-EGFP-GPI_DAF_, and TST-EGFP-GPI_CEAM7_, had higher enrichment, with the positive cell ratio elevated from 16%, 17%, and 19%–75%, 78%, and 59%, respectively (Figures 3A,B).

In addition, we calculated the positive cell enrichment fold to represent the ability of the six tags in cell sorting. The results showed that the three GPI type sorting tags had higher enrichment fold than the TMD type sorting tags in all the three cell lines, including K-562, Lenti-X 293T, and 22Rv1 cells (Figure 3C).

During the cell sorting, we noticed that when we put the tube on a magnetic stand to separate the bead/cell complexes, they ran suddenly and roughly toward the magnet, which might cause the dropping of the bound positive cells. So we tried to separate the cell/magnetic beads by free settling instead of applying an external magnetic field, and obtained an higher positive cell percentage. In the case of cell sorting with the TST-EGFP-GPI_BY55_ tag in K-562 cells, the positive cell ratio reached up to 95% (Figure 3D).

**FIGURE 4:**
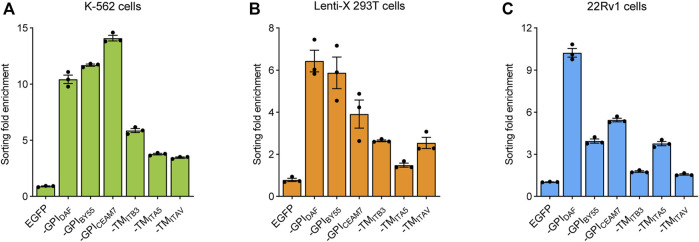
Cell sorting fold enrichment of the EGFP expression. Bar chart showing the enrichment fold of EGFP RNA expression level after cell soring using six sorting tags in K-562 **(A)**, Lenti-X 293T **(B)**, and 22Rv1 cells **(C)**, respectively. The fold enrichment represents the change of the β-actin reference gene-normalized EGFP expression level after cell sorting. The cells transfected with pEGFP-C2 plasmids were used as the negative control. The values come from three RT-qPCR replicates.

In addition, we determined the cell sorting enrichment fold with EGFP expression at the RNA level. The RNA was extracted from the transfected cells before or after cell sorting, and the EGFP expression was measured using RT-qPCR with the β-actin gene as a control. We found that all the six sorting tags could efficiently enrich the transfection-positive cells and lead to a dramatically higher EGFP expression level in the resulted cells. Consistent with the flow cytometry analysis results, the three GPI type sorting tags, TST-EGFP-GPI_BY55_, TST-EGFP-GPI_DAF_, and TST-EGFP-GPI_CEAM7_, had higher enrichment fold than the three TMD type ones in K-562 (Figure 4A), Lenti-X 293T (Figure 4B), and 22Rv1 cells (Figure 4C). The three GPI type sorting tags worked more effectively and reached an enrichment fold of over ten times in K-562 cells (Figure 4A). In the other suspension cells, Jurkat, the enrichment folds of the three GPI type sorting tags were also over nine times (Supplementary Figure S1C). Noticeably, the enrichment fold of the six sorting tags had a big fluctuation in the Lenti-X 293T and 22Rv1 cells (Figures 4B,C). It indicated that detaching and resuspending operation of the adherent cells might disturb cell sorting. Given the higher and more stable cell sorting enrichment fold, we choose the TST-EGFP-GPI_BY55_ sorting tags for further evaluation and application.

### Cell Sorting Helps Gene Overexpression Analysis

**FIGURE 5:**
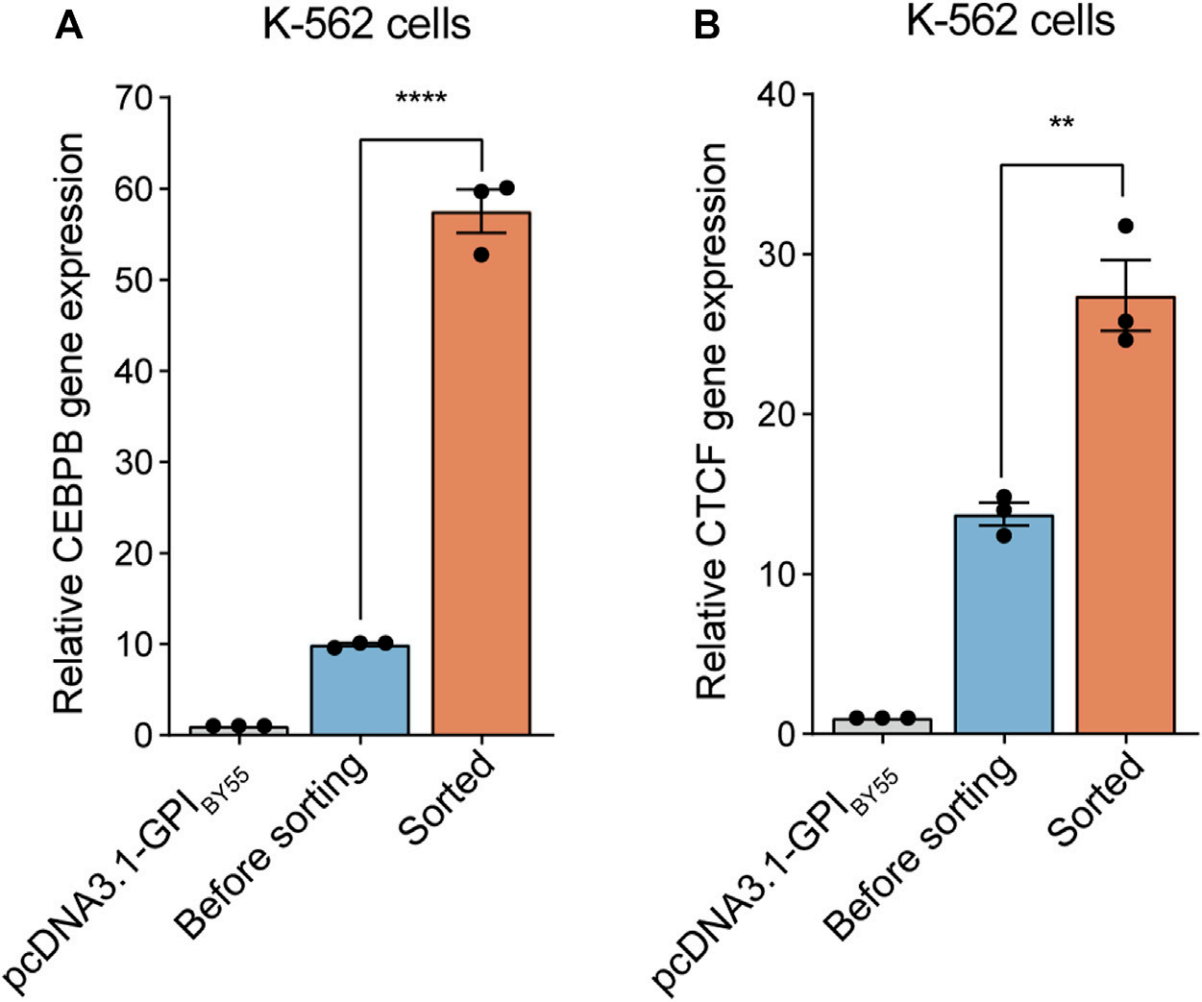
Application of cell sorting in gene overexpression. Bar chart representing *CEBPB* **(A)** and *CTCF* **(B)** gene expression level in K-562 cells overexpressing corresponding gene enriched or not by cell sorting. The cells transfected with pcDNA3.1-GPI_BY55_ blank vector were included for comparison. Values represent *CEBPB* or *CTCF* expression level normalized to the *β-actin* reference gene, determined using RT-qPCR experiments. Error bars, means ± SEM of three independent experiments. **p < 0.01, and ****p < 0.0001 as determined by an unpaired, two-tailed Student’s t-test.

We first tested the cell sorting system in a gene overexpression experiment. The coding sequence of the TST-EGFP-GPI_BY55_ was inserted into the pcDNA3.1 vector in place of the neomycin resistance gene to obtain the pcDNA3.1-GPI_BY55_ sorting vector for gene overexpression (Supplementary Figure S3B). The vector could effectively drive the expression and membrane targeting of the sorting tag (Supplementary Figure S2A). Then we cloned two transcription factor genes, *CEBPB* and *CTCF*, from K-562 cDNA into this vector and transfected K-562 cells. The expression level of the target genes in transfected cells before or after affinity sorting was determined by RT-qPCR. We observed that cell transfection increased the *CEBPB* gene expression level by ten times the control, and affinity cell sorting operation dramatically increased the expression level by up to 58 times in the sorted cells (Figure 5A). Similarly, cell transfection with CTCF expression plasmid increased the mRNA level of the *CTCF* gene by 14 times that of the endogenous gene, and further affinity cell sorting increased the expression level effectively by 27 times (Figure 5B). It indicates that the affinity cell sorting system can greatly help the gene overexpression study.

### Cell Sorting Assists shRNA Gene Knockdown Assay

Furthermore, we investigated the affinity cell sorting system in the shRNA gene knockdown assay. The encoding sequence of TST-EGFP-GPI_BY55_ was cloned into the pLKO.1 vector in the place of the puromycin resistance gene to construct a plasmid for gene knockdown assay (Supplementary Figure S3C). Fluorescence microscopy showed that the affinity sorting vector could effectively express the EGFP sorting tags and locate them to the cell surface (Supplementary Figure S2B).

**FIGURE 6:**
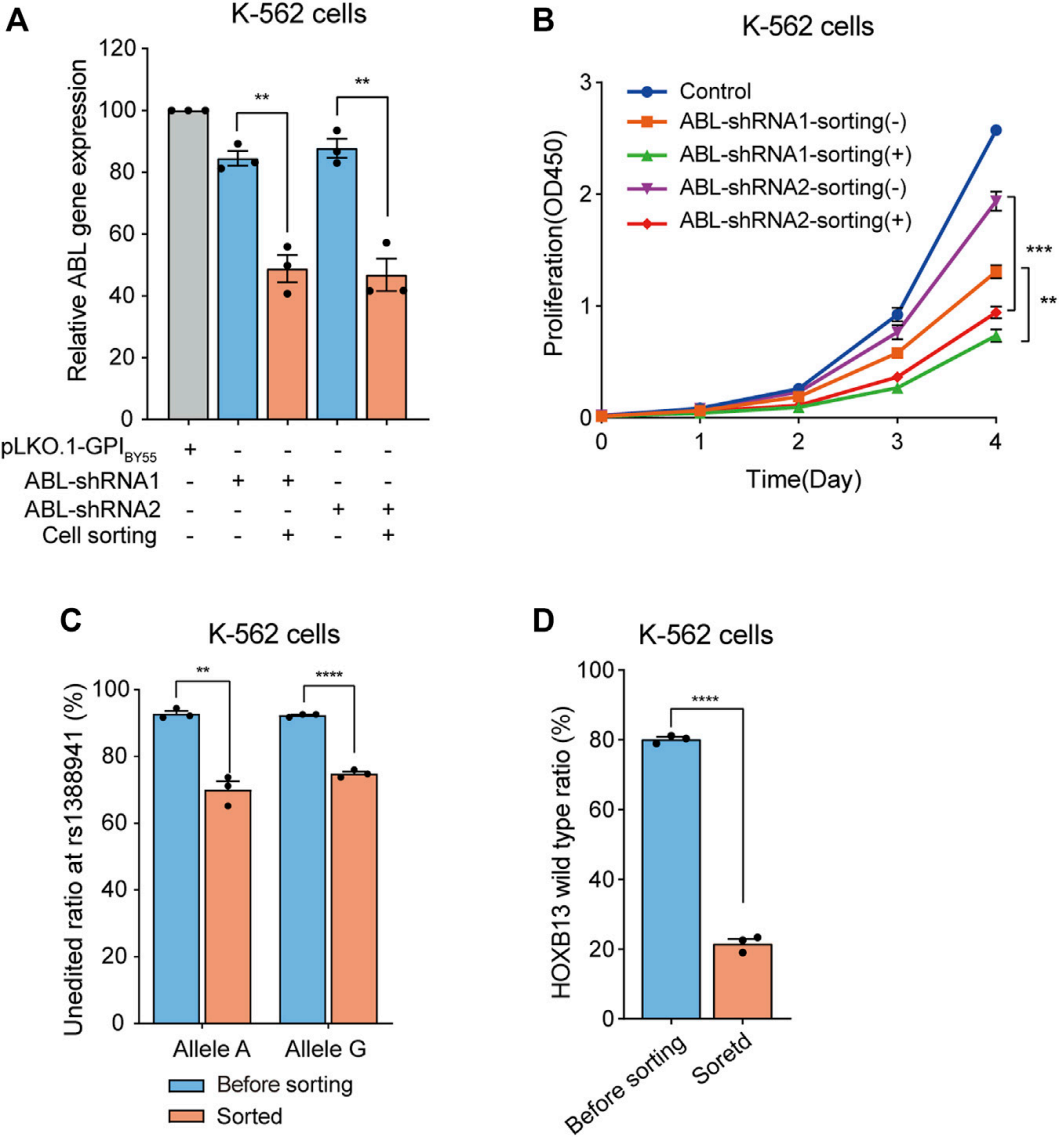
Application of cell sorting in shRNA knockdown and CRISPR/eCas9 gene editing. **(A)** Bar chart showing the relative *ABL* gene expression level in K-562 cells transfected with the pLKO.1-GPI_BY55_ vector expressing ABL-shRNA1 and ABL-shRNA2, enriched or not by cell sorting. Values are from three RT-qPCR experiments. **(B)** The cell proliferation analysis of the K-562 cells expressing ABL-shRNA1 or ABL-shRNA2, with or without enrichment by affinity cell sorting, measured using the CCK-8 kit. Data from three biological replicate wells. **(C)** Bar chart showing genome editing frequency at rs1388941 locus in K-562 cells transfected with CRISPR/eCas9-GPI_BY55_ vector encoding sgRNA targeting rs1388941 region, with or without enrichment by cell sorting. The values represent the allele-specific unaltered gene percentage from three getPCR experiments. **(D)** Genome editing in K-562 cells using CRISPR/eCas9-GPI_BY55_ vector encoding the *HOXB13* gene sgRNA. The values represent the unaltered gene percentage from three getPCR experiments. Error bars, means ± SEM. **p < 0.01, ***p < 0.001, and ****p < 0.0001 as determined by an unpaired, two-tailed Student’s t-test.

Then we constructed two shRNA expression plasmids targeting the *BCR-ABL* fusion gene, a vital marker molecule of chronic myelogenous leukemia (CML) (Quintas-Cardama and Cortes, 2009; Salem et al., 2017), encoding a continuously activated tyrosine kinase activity that leads to overproliferation (Reckel et al., 2017). The K-562 cells express a high level of endogenous *BCR-ABL* fusion gene (Reckel et al., 2017; Antonenko and Telegeev, 2020). We found that transfection of the two shRNA plasmids, ABL-shRNA1 and ABL-shRNA2, in K-562 cells could down-regulate the ABL expression slightly, with knockdown efficiency of 15% and 12%, respectively. Strikingly, in the cells enriched by affinity sorting, the knockdown efficiency of ABL-shRNA1 and ABL-shRNA2 dramatically increased to 51% and 53%, respectively (Figure 6A).

Studies have shown that reducing the BCR-ABL expression can inhibit the proliferation of leukemia cells (Szczylik et al., 1991; Skorski et al., 1993; Liu et al., 2021). Hence, we evaluated the proliferation ability of the shRNA transfected cells before or after affinity cell sorting using the CCK-8 kit. The results showed that both ABL-shRNA1 and ABL-shRNA2 transfection significantly inhibited cell proliferation (Figure 6B). Noticeably, a more significant inhibition effect was observed in the cells enriched in the affinity cell sorting (Figure 6B). It indicates that the transfection positive-cell affinity sorting system can deeply assist gene knockdown experiments.

### Cell Sorting for Genome Editing

The CRISPR/Cas9 system has been widely used in gene function research and the treatment of genetic diseases (Jinek et al., 2012; Cong et al., 2013; Mali et al., 2013). Increasing the editing positive cell ratio is also pivotal in genome editing research. Here, we also evaluated the affinity cell sorting system in the genome editing experiment. The expression unit of the TST-EGFP-GPI_BY55_ sorting tag was cloned into the high-fidelity eCas9 (Kleinstiver et al., 2016; Li et al., 2019a) expression vector and constructed an affinity sorting vector for gene editing (Supplementary Figure S3D). Fluorescence microscopy analysis showed that cells transfected with the vector could effectively express the EGFP sorting tags and localize them to the cell membrane (Supplementary Figure S2C).

The function and mechanism researches of the disease risk-associated SNPs play pivotal roles in genetic pathology (Huang et al., 2014; Gao et al., 2018; Ma et al., 2021; Ren et al., 2021). Modifying risk SNP sites through genome editing to obtain risk SNP cell models is essential in the study. Here, we chose rs1388941, a risk SNP site associated with osteonecrosis in children with acute lymphoblastic leukemia (Karol et al., 2015), as an example for the genome editing study and designed a sgRNA targeting the neighbor region. We transfected K-562 cells, A/G heterozygous at the SNP site, and performed affinity cell sorting to enrich the transfection-positive cells. After cell sorting, genomic DNA was extracted, and the unedited ratio at both alleles was determined through the getPCR method (Li et al., 2019a). We found that the editing ratio at both alleles was only about 7% before sorting. Remarkably, the editing ratio significantly increased to about 30% and 25% (Figure 6C) in enriched cells.

To better manifest the potential of the affinity cell sorting system in genome editing experiment, we further performed genome editing using a sgRNA that targeting the *HOXB13* gene with a high editing activity in Lenti-X 293T cells (Li et al., 2019b). Analysis in K-562 cells showed that cell sorting significantly improved the editing efficiency at this sgRNA target from 20% to 79% (Figure 6D). It indicates that the transfection-positive cell sorting system can promote genome editing experiments with hard-to-transfect cells.

### Universal Sorting Plasmid Co-Transfection Permits Efficient Positive-Cell Sorting

Presently, cloning the target genes in the given vectors encoding the sorting marker is usually obligatory for positive cell sorting. To break this limit, we investigated the strategy of co-transfecting the aim plasmid with a universal plasmid expressing the sorting tag, which will allow the sorting system to be easily transplanted to any other experiments in need. Remarkably, this strategy will exempt the need to construct a vector for a given type of application, and experiments can start directly with the existing plasmid.

**FIGURE 7:**
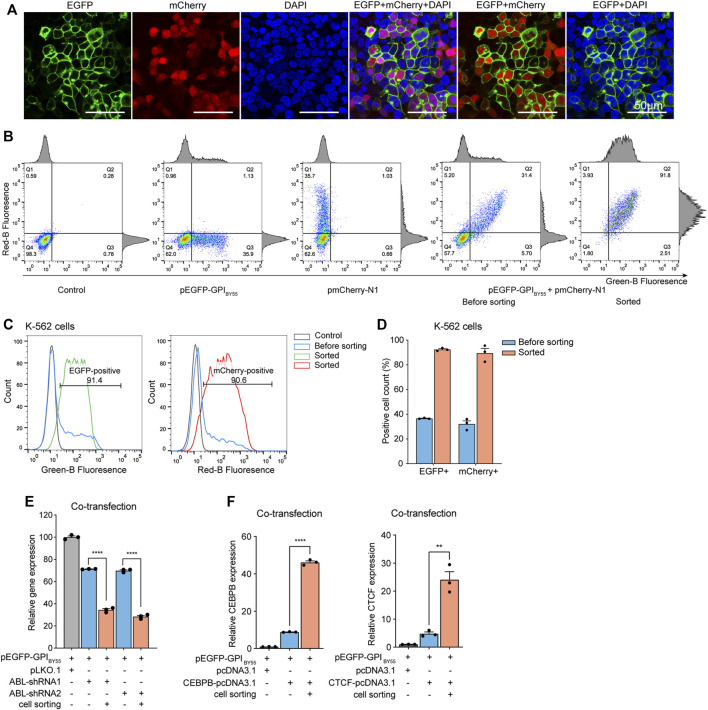
The sorting plasmid co-transfection strategy. **(A)** Laser scanning confocal microscopy of Lenti-X 293T cells co-transfected with the pEGFP-GPI_BY55_ and pmCherry-N1 plasmids. DAPI stains cell nuclei. Cells were observed at ×200 magnification, scale bar = 50 μm. **(B)** Flow cytometry density dot plot of the K-562 cells co-transfected with the pEGFP-GPI_BY55_ and pmCherry-N1 plasmids with or without enrichment by cell sorting. Cells transfected with pEGFP-GPI_BY55_ or pmCherry-N1 were used as fluorescence compensation controls. The percentage of cells in each quadrant is represented. Q1: EGFP^−^/mCherry^+^; Q2: EGFP^+^/mCherry^+^; Q3: EGFP^+^/mCherry^−^; Q4: EGFP^−^/mCherry^−^. The corresponding flow cytometry histograms on the border represent cell counts vs. fluorescence intensity for green or red channels. **(C)** Flow cytometry histograms showing the normalized cell counts vs. fluorescence intensity for the green and red channels. The gray line represents the untransfected negative control K-562 cells, the blue line represents the K-562 cells co-transfected with pEGFP-GPI_BY55_ and pmCherry-N1, and the green/red lines represent the co-transfected cells enriched by cell sorting. **(D)** The bar chart showing the percentage of fluorescence positive cells for EGFP and mCherry, respectively, in the co-transfected K-562 cells with or without enrichment by cell sorting. Values are from three biological replicates. **(E)** The relative *ABL* gene expression level in K-562 cells co-transfected with pEGFP-GPI_BY55_ sorting plasmid and ABL-shRNA1 or ABL-shRNA2, with or without enrichment by cell sorting. Values are from three RT-qPCR experiments with β-actin as the reference gene. **(F)** The relative gene expression level of *CEBPB* and *CTCF* genes in K-562 cells co-transfected with pEGFP-GPI_BY55_ sorting plasmid and *CEBPB* or *CTCF* overexpression plasmid, respectively, with or without enrichment by cell sorting. Values are from three RT-qPCR experiments with β-actin as the reference gene. Error bars, means ± SEM. Two-tailed *t*-test, **p < 0.01 ****p < 0.0001.

We co-transfected Lenti-X 293T cells with the TST-EGFP-GPI_BY55_ expression plasmid and the pmCherry-N1 in equal proportion. Laser confocal microscopy analysis showed that the EGFP and the mCherry displayed an obvious co-positive pattern (Figure 7A). The positive cells for EGFP and mCherry accounted for 52% and 48%, respectively. Remarkably, EGFP and mCherry double-positive cells accounted for up to 44% (Supplementary Figure S4).

Furthermore, we performed similar co-transfection on K-562 cells and applied affinity cell sorting to enrich the transfection-positive cells. Flow cytometry analysis showed that EGFP and mCherry displayed a prominent co-positive feature again (Figures 7B–D). In the co-transfected K-562 cells, the proportions of EGFP positive cells and mCherry positive cells were 36% and 32%, respectively. Remarkably, the double-positive cells accounted for up to 29% (Figure 7B). In cells enriched by affinity cell sorting, the proportion of EGFP positive, mCherry positive, and double-positive cells reached 93%, 90%, and 86%, respectively (Figures 7B–D). It indicates that co-transfection with a universal sorting plasmid can allow effective affinity cell sorting to enrich the positive cells containing the target plasmid.

Interestingly, in the co-transfected K-562 cells, the ratio of EGFP single positive, mCherry single positive, and EGFP/mCherry double-positive cells was approximately 1:1:6. Assumed that each liposome microdroplet carries multiple plasmid molecules and the plasmids of similar size have equal opportunity to enter cells, if three plasmids enter one cell, the corresponding ratio is supposed to be 1:1:6. Thus, we proposed that an average of three plasmids entered each cell under the transfection conditions.

### Sorting Plasmid Co-Transfection Assists Gene Knockdown and Overexpression Experiments

Next, we co-transfected the pEGFP-GPI_BY55_ plasmid with the pLKO.1 plasmid expressing ABL shRNA into K-562 cells and performed affinity cell sorting. RT-qPCR analysis showed that ABL-shRNA1 and ABL-shRNA2 downregulated *ABL* gene expression by 29% and 31%, respectively. Noticeably, in the cells enriched by affinity sorting, the knockdown efficiencies of ABL-shRNA1 and ABL-shRNA2 increased dramatically to 66% and 72%, respectively (Figure 7E), comparable to the levels of the single-plasmid transfection experiment.

Further, we applied the sorting plasmid co-transfection strategy in gene overexpression experiments. The pEGFP-GPI_BY55_ plasmids were co-transfected with pcDNA3.1 vector encoding *CEBPB* or *CTCF* gene into K-562 cells. RT-qPCR analysis showed that the transfection increased the expression levels of CEBPB and CTCF by nine times and five times, respectively. While in the sorted cells, the expression levels of CEBPB and CTCF dramatically increased to 46 times and 24 times, respectively (Figure 7F), comparable to that of the single-plasmid transfection experiments. It indicates that the sorting plasmid co-transfection strategy can effectively enrich positive cells through affinity cell sorting and benefit the gene knockdown and gene overexpression assays to an extent comparable to the single plasmid transfection strategy.

## Discussion

We developed a gene transfer-positive cell sorting system to help gene studies, especially those in hard-to-transfect cells. The system is based on a fluorescent affinity sorting tag designed by fusing EGFP with an N-terminal TST peptide and a GPI signal module from the BY55 gene. The positive cells expressing the sorting tags on the cell surface can bind Strep-Tactin magnetic beads and hence are enriched effectively. Besides, the EGFP module of the sorting tag enables the positive cells to be evaluated through fluorescence microscopy and flow cytometry. Furthermore, we demonstrated the great potential of the sorting system in a series of gene function studies, including gene overexpression, shRNA knockdown, and genome editing.

Our cell sorting system has several advantages compared to the existing magnetic cell sorting methods. Firstly, we use a GPI anchor-linked EGFP molecule as the basis to construct the sorting tag, which less probably brings about a disturbance on the cell signaling and gene function compared to the truncated LNGFR (Matheson et al., 2014) and mouse H-2K^k^ (Wei et al., 2001) molecules used in previous methods. Secondly, the TST is used as the affinity ligand to display on the cell surface and enable affinity cell sorting using the Strep-Tactin® or Strep-Tactin®XT magnetic beads. The nM or even pM level affinity of the TST for the receptor makes the system more efficient to pull out the positive cells than the SBP tags (Matheson et al., 2014), and the H-2K^k^ (Wei et al., 2001) molecules do. Thirdly, the EGFP module in our sorting tag allows convenient evaluation of the positive cells through fluorescent microscopy and flow cytometry, which is difficult for the existing MACS methods. Fourthly, the GPI anchoring structure displaying the sorting tag on the cell surface in our system is obviously more efficient than the transmembrane domain used in the existing MACS methods.

Compared with the drug screening methods, our sorting system is more time-saving and efficient because of its better versatility for different cell lines and the ability to realize transfection-positive cells enrichment with a one-step affinity sorting operation. Correspondingly, the drugs usually take several days to kill the transfection-negative cells, and on the other hand, pre-experiments are obligatory to explore the working drug concentration for different cell lines. Meanwhile, our sorting system is supposed to cause fewer side effects on the cell function because it does not bring about cell toxicity as the screening drugs do. Unlike the restricted application in adherent cells for the drug screening methods, our sorting system is applicable to both adherent and suspension cells.

Compared to the FACS method, our cell sorting system also has several advantages. Firstly, we do not require equipment like the cell sorter, which is expensive and not readily available in most laboratories. Secondly, the throughput of our cell sorting system is easy to expand by using more affinity beads and can be performed in parallel. Whereas, even the cutting-edge cell sorter, the sorting speed is still limited, resulting in prolonged sorting time if many cells are demanded. Thirdly, our cell sorting operation applies a more mild operation which will allow less mechanical damage to the cells than the FACS method.

Finally, our gene transfer-positive cell sorting system displayed great potential to enrich gene transfection positive cells in gene study applications, including gene overexpression, gene shRNA knockdown, and genome editing. In the future, the application can be easily expanded to other gene study fields by simply inserting the expression cassette of TST-EGFP-GPI_BY55_ into the target vectors. Furthermore, we can further expand the versatility of the cell sorting system by replacing the EGFP with other fluorescent proteins such as mCherry, dsRed, RFP, YFP, and BFP, and replacing the TST tags can with other affinity tags such as CBP (calmodulin-binding peptide), MBP (maltose-binding protein), and His-Tag protein tags. In addition, the sorting system should work well in a wide range of biological systems with GPI anchoring systems, including most eukaryotes and some Archaeobacteria (Yadav and Khan, 2018; Nakano et al., 2021).

Notably, the co-transfection experiment of the TST-EGFP-GPI_BY55_ expression plasmid and pmCherry-N1 plasmid manifested a strongly co-positive character for the two fluorescence. This property permits the gene transfer-positive cell sorting in a more simple but efficient way by co-transfecting existing vectors with the universal TST-EGFP-GPI_BY55_ expression plasmid. More interestingly, the co-transfection strategy can be further expanded to other transfection-positive cell sorting systems such as FACS and MACS. The co-transfection strategy can avoid the trouble of reconstructing the target gene plasmid on the sorting vector, allow the target plasmid to accommodate larger insertion, and hence lead to reduced time and manpower cost. In summary, the gene transfer-positive cell sorting system possesses great potential to burst gene function study in hard-to-transfect cells.

## Materials and Methods

### Plasmid Construction and Gene Cloning

The coding sequences of the six membrane location signals were synthesized in the pUC57a vector (GENEWIZ Co., Suzhou, China) and amplified separately through PCR. The EGFP coding sequence was amplified from the pEGFP-C2 plasmid, and the Twin-Strep-Tag (TST) coding sequence containing 30 amino acid residues (WSHPQFEK-GGGSGGGSGGS-SAWSHPQFEK) was obtained by primer self-PCR. Then the above PCR products were mixed at the mole ratio of 1:1:1:1 and subjected to overlapping PCR to obtain the whole length sorting tag coding sequence. The resulted PCR products were digested by FastDigest AgeI and FastDigest BglII (Thermo Fisher, Waltham, MA, United States) and then joined with the FastDigest AgeI/FastDigest BglII linearized pEGFP-C2 vector to obtain the expression vectors for the six affinity sorting tags (Supplementary Figure S3A). Primer sequences are shown in Supplementary Table S3.

The coding sequence of TST-EGFP-GPI_BY55_ was amplified from the pEGFP-GPI_BY55_ vector with flanking homology arm sequences and cloned into the PCR-linearized pcDNA™ 3.1/V5-HIS A (Invitrogen, Carlsbad, CA, United States) vector in place of the neomycin coding sequence using the ClonExpress II One-step Cloning Kit (C112, Vazyme, Nanjing, China) to obtain the affinity sorting plasmid for gene overexpression (Supplementary Figure S3B). The TST-EGFP-GPI_BY55_ coding sequence was also amplified by PCR. The PCR product was digested with FastDigest BamHI and FastDigest KpnI (Thermo Fisher, Waltham, MA, United States) and ligated with the BamHI/KpnI linearized pLKO.1 vector (Sigma, St. Louis, MO, United States) using T4 DNA Ligase (EL0011, Thermo Fisher, Waltham, MA, United States) to obtain the affinity sorting vector for U6-shRNA expression (Supplementary Figure S3C). The whole expression cassette of the Nsig-TST-EGFP-GPI_BY55_ was PCR amplified and joined with NotI linearized pX330 vector (#42230, Addgene) (Cong et al., 2013) expressing eCas9 (R661A/Q695A/Q926A) (Kleinstiver et al., 2016; Li et al., 2019a) using the ClonExpress II One-step Cloning Kit (C112, Vazyme, Nanjing, China) to obtain the affinity sorting vector for genome editing experiments (Supplementary Figure S3D). The related primer sequences are shown in Supplementary Tables S4–S6.

For constructing the CEBPB and CTCF overexpression plasmids, the corresponding CDS sequences were amplified from K-562 cDNA using primers listed in Supplementary Table S4, digested with FastDigest HindIII and XbaI (Thermo Fisher, Waltham, MA, United States), and then joined to HindIII/XbaI linearized pcDNA3.1-GPI_BY55_ or pcDNA3.1 vector with T4 DNA ligase (EL0011, Thermo Fisher, Waltham, MA, United States). For constructing the ABL shRNA expression plasmids, annealed oligos (Supplementary Table S5) bearing ABL-shRNA1 (Sigma, St. Louis, MO, United States; #TRCN0000039898) or ABL-shRNA2 (TRCN0000039901) were inserted into the AgeI/EcoRI linearized pLKO.1-GPI_BY55_ or pLKO.1 vector. To construct the genome editing plasmid targeting the rs1388941 locus, we designed the CRISPR target sequence on the CRISPRdirect website (http://crispr.dbcls.jp/). The sgRNA sequence of *HOXB13* gene was designed and verified by laboratory previously (Li et al., 2019b). The annealed oligos (Supplementary Table S6) bearing the gRNA sequence were inserted into BbsI linearized CRISPR/eCas9-GPI_BY55_ vector using T4 DNA ligase. All plasmids for cell transfection were extracted with an Endo-Free Plasmid Mini Kit I (D6948-02, OMEGA, Guangzhou, China) and purified by ethanol precipitation.

### Cell Culture

The Lenti-X 293T cells were purchased from Clontech (#632180) and cultured in DMEM (Gibco, New York, NY, United States) medium. The 22Rv1 (CRL-2505) cells and K-562 cells were purchased from ATCC and maintained in RPMI 1640 (Gibco, New York, NY, United States) and IMDM (Gibco, New York, NY, United States) medium, respectively. All media were supplemented with 1% antibiotics (penicillin and streptomycin, Sigma, St. Louis, MO, United States) and 10% fetal bovine serum (Gibco, New York, NY, United States). Cells were kept at 37°C and 5% CO_2_ and generally subcultured every 2–3 days, regularly tested for *mycoplasma* using mycoblue®*mycoplasma* Detector (D101-02, Vazyme, Nanjing, China).

### Confocal Laser Scanning Microscopy

The Lenti-X 293T cells were inoculated on a glass slide in a 24-well plate at a density for cells to reach 70% at transfection. On the next day, 0.8 µg pEGFP-C2, pEGFP-GPI_BY55_, pEGFP-GPI_DAF_, pEGFP-GPI_CEAM7_, pEGFP-TM_ITB3_, pEGFP-TM_ITA5_ and pEGFP-TM_ITAV_ plasmids were transfected using 1×PEI reagent (#408727, Sigma, St. Louis, MO, United States) as described previously (Ma et al., 2021) with a DNA: PEI ratio of 1:1.5. Forty-eight hours post-transfection, cells were washed twice with 1×PBS and fixed with 4% paraformaldehyde at room temperature for 10–15 min in the dark and then gently washed twice with 1×PBS. Then, the nuclei were counterstained with 10 μg/ml DAPI (4′, 6-diamidine-2-phenylindole, C0060, Solarbio, Beijing, China) reagent according to the instructions by 15 min incubation at 37°C followed by twice washing with 1×PBS. The slides were sealed with Antifade Mounting Medium (S2100, Solarbio, Beijing, China) and stored in a wet box in the dark. The cells were observed under an LSM900 Super Resolution Laser Scanning Confocal Microscope (ZEISS, Oberkohen, baden-Wurberg, Germany).

### Cell Transfection and Affinity Cell Sorting

For Lenti-X 293T and 22Rv1, cells were inoculated in 6-well plates and transfected on the next day as described previously (Ma et al., 2021) when reaching a 50–70% confluence. Briefly, 1.0 µg or 1.2 µg plasmid was mixed with polyethyleneimine reagent (PEI, #408727, Sigma, St. Louis, MO, United States) with a DNA: PEI ratio of 1:1.5 and applied to Lenti-X 293T and 22Rv1 cells, respectively. The cells were subjected to fluorescent microscopy or affinity cell sorting 36–48 h later. For K-562, 3.5×10^5^ cells were inoculated in 6-well plates and directly transfected with 1.5 µg plasmid using Lipofectamine 2000 (11668-019, Invitrogen, Carlsbad, CA, United States) transfection reagent at a ratio of 1:3, according to the Lipofectamine 2000 Transfection Reagent Protocol.

The cells were applied to affinity cell sorting 36–48 h post-transfection. For adherent Lenti-X 293T and 22Rv1 grown in 6-well plates, cells were rinsed with 500 µl 1×PBS solution and dispersed into a single-cell suspension through incubation with 500 µl 1× Non-enzymatic Cell Dissociation Solution (C5914, Sigma, St. Louis, MO, United States) for 5–10 min. The cells were collected and washed twice with 1×PBS solution and then resuspended in 250 µl binding buffer (IMDM with 2% FBS). For suspension Jurkat and K-562, cells were collected directly, washed twice with 1×PBS solution, and then resuspended in 250 µl binding buffer.

For each cell sorting reaction, 100 µl BeaverBeads™ Magrose Strep-Tactin (#70808, Beaver, Suzhou, China) were washed twice, resuspended in 250 µl binding buffer, and then mixed with the cell suspension gently. The mixture was placed on a rotating mixer and incubated at 10 rpm for 15 min at room temperature. Then the magnetic beads were separated by staying on a magnetic rack for 2 min. After removing the supernatant, the beads/cells complexes were gently rinsed twice with IMDM medium without FBS. Finally, the gene transfection-positive cells captured on the magnetic beads were released in 300 μl complete medium by rotating at 15 rpm for 5 min. The magnetic beads were separated on the magnetic rack to collect the supernatant containing transfection-positive cells.

For the cell sorting strategy separating the beads/cells complex by free settling, 1.5 ml D-PBS (E607009, Sangon Biotech, Shanghai, China) solution containing 0.1% BSA (A600332, Sangon Biotech, Shanghai, China) was used as the binding buffer and washing buffer to prepare cell suspension and beads. After incubation on a rotating mixer, the beads/cells complexes were separated from the unbound cells by free settling for 1 min, utilizing their density difference. The bead/cell complexes were resuspended in 1.5 ml D-PBS solution containing 0.1% BSA, and the supernatant was removed after 1 min free settling. The transfection-positive cells captured on the beads were collected in a D-PBS solution containing 0.1% BSA or a specific cell medium dependent on the downstream applications.

### Flow Cytometry

Cells from the affinity cell sorting experiment were directly subjected to flow cytometry analysis on a NovoCyte (ACEA Biosciences, San Diego, California, United States) or Guava easyCyte (Luminex, Austin, Texas, United States). The default detector gain was used for FSC and SSC, while the detector gain of FITC was adjusted to locate the negative control cell peak around 1× 10^2^ and 1×10^3^. For each analysis, 10,000 events were acquired at a low-speed flow rate. In multicolor fluorescence analysis of EGFP and DsRed transfected cells, fluorescence compensation was performed using compensation control cells that were transfected with EGFP and DsRed plasmids separately.

### RNA Extraction and Quantitative RT-PCR

RNA samples were prepared using the GeneJET RNA Purification Kit (K0732, Thermo Scientific, Waltham, MA, United States) according to the product user guide. Residual genomic DNA was removed by the RapidOut DNA Removal Kit (K2981, Thermo Scientific, Waltham, MA, United States) according to the user guide. RNA was reverse transcribed into cDNA with the High-capacity cDNA Reverse Transcription Kit (4368813, Applied Biosystems, Waltham, MA, United States) using the accompanied random primers, following the product manual. The cDNA products were used directly for subsequent experiments or stored in a −80°C refrigerator.

The mRNA expression levels of EGFP, ABL, CEBPB, or CTCF in the transfected cells before or after cell sorting were determined by qPCR using Taq388 mix (Du et al., 2022) on a QIAGEN Q-Rex machine as previously described (Ma et al., 2021) with primers listed in Supplementary Table S6. Each pair of PCR primers were tested, and primers with good specificity and amplification efficiency were selected for quantitative PCR analysis. The endogenous *ACTB* (β-actin) gene expression was used for normalization. The enrichment fold of the EGFP mRNA for the cell sorting was calculated to characterize the cell sorting efficiency of each sorting tag. In the gene overexpression and knockdown experiment, the empty vector was used as the control in the transfection.

### getPCR

The genomic DNA was prepared from transfected cells 48 h post-transfection with or without cell sorting, using TIANamp genomic DNA kit (#DP304-03, Tiangen Biological Technology, Beijing, China). The genome-editing efficiency was evaluated using the getPCR method (Li et al., 2019a) with primers listed in Supplementary Table S6 that had been evaluated for the amplification efficiency and specificity. The qPCR was performed using the Taq 388 mix (Du et al., 2022) on a QIAGEN Q-Rex machine with the program: 5min initial denaturation at 95°C, then 40 cycles of 95°C for 30 s, 67°C for 30 s and 72°C for 15 s with fluorescence acquirement, followed by a final melting curve step increasing from 65°C to 95°C.

### Cell Proliferation Assays

K-562 cells transfected with ABL shRNA with or without enrichment through affinity cell sorting were dispersed into single-cell suspension and inoculated in a 96-well plate at a density of 1000 cells per well. K-562 cells transfected with the pLKO.1-GPI_BY55_ blank plasmid were also inoculated as the control. At 0, 24, 48, 72, and 96 h post-seeding, 1/10 volume CCK-8 reagent (Cell Counting Kit-8, MA0218, Meilun, Dalian, China) was added to the cells, and OD450 nm was acquired on a microplate reader after 3-h incubation, with 600 nm as the reference wavelength. Values were obtained from three independent replicate wells, and the statistical significance was calculated using a two-tailed Student’s t-test.

## Data Availability

The original contributions presented in the study are included in the article/Supplementary Material, further inquiries can be directed to the corresponding author.
