# Supplementary material for: A Gene Transfer-Positive Cell Sorting System Utilizing Membrane-Anchoring Affinity Tag
Source: Front Bioeng Biotechnol. 2022 Jun 16;10:930966. doi: 10.3389/fbioe.2022.930966 (PMC9244562; doi:10.3389/fbioe.2022.930966)
Supplement: Supplementary file 1 [file DataSheet1.PDF]

Supplementary Material

1. Supplementary Figures

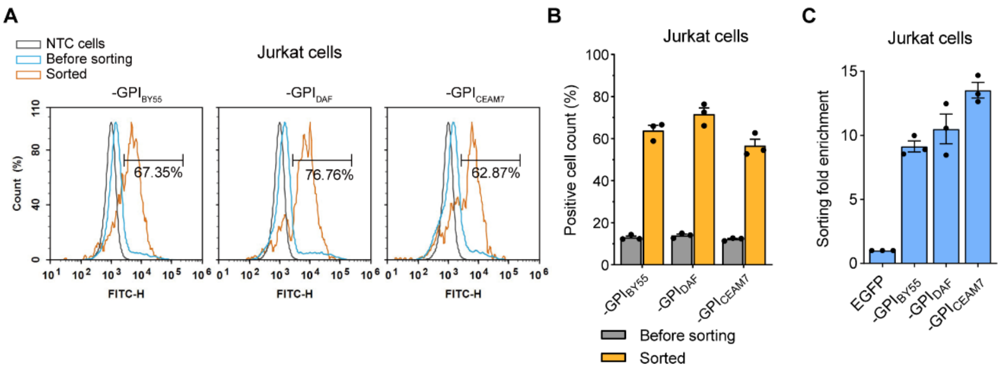

Supplementary Figure 1. Flow cytometry analysis of Jurkat cells from cell sorting. (**A**) Flow cytometry histograms of Jurkat cells transfected with three GPI-based sorting tag plasmids, enriched or not by cell sorting. The normalized Histogram is shown as a percentage value that represents the maximum value of the layer histogram. The gray layer represents the control cells without transfection, and the blue and the orange layer represent the before sorting or sorted cells respectively. The percentage of fluorescence-positive cells in the enriched cells is represented. (**B**) Bar chart showing the percentage of fluorescence positive cells in the sorted Jurkat cells determined by flow cytometry analysis. Values are from three independent biological replicates. (**C**) Bar chart showing the enrichment fold of EGFP RNA expression level after cell soring in Jurkat. The fold enrichment represents the change of the β-actin reference gene-normalized EGFP expression level after cell sorting. The cells transfected with pEGFP-C2 plasmids were used as the negative control. The values from three RT-qPCR replicates.

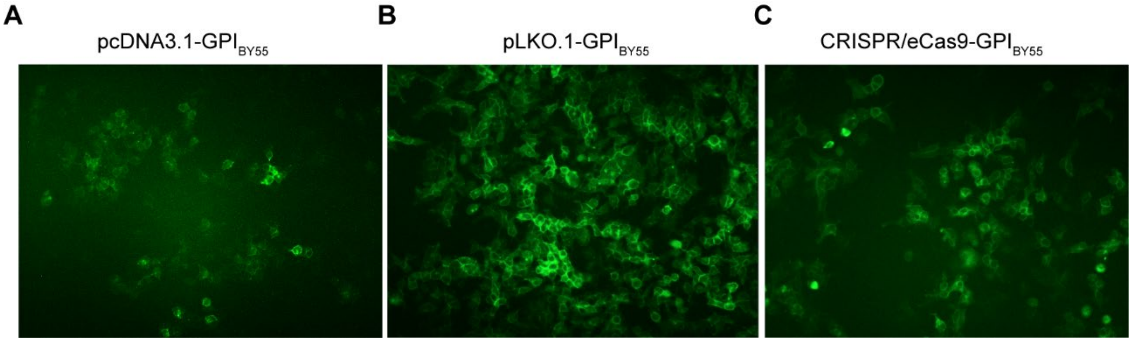

Supplementary Figure 2. Fluorescent microscopy analysis of TST-EGFP-GPI$_{BY55}$ sorting tag. The fluorescent microscopy of the Lenti-X 293T cells transfected with (**A**) pcDNA3.1-GPI$_{BY55}$, (**B**) pLKO.1-GPI$_{BY55}$ and (**C**) CRISPR/eCas9-GPI$_{BY55}$ vectors, respectively.

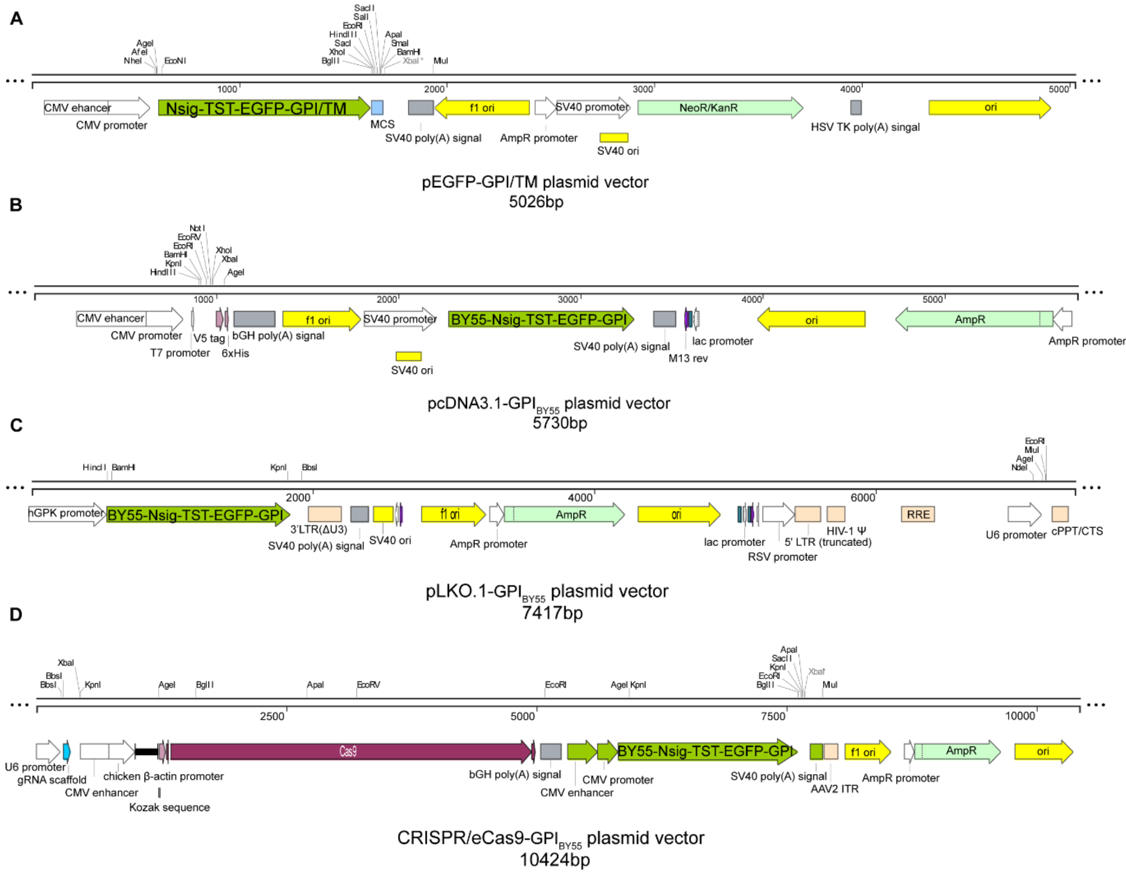

Supplementary Figure 3. Plasmid profiles. (**A**) Schematic diagram of six sorting tag plasmids based on pEGFP-C2 vector. Schematic diagram of TST-EGFP-GPI$_{BY55}$ sorting tag plasmid constructed on the pcDNA3.1 (**B**), pLKO.1 (**C**) and CRISPR/eCas9 (**D**) vectors.

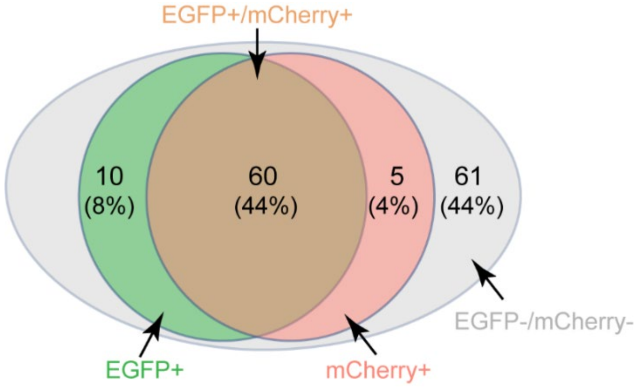

Supplementary Figure 4. Quantitative analysis of the Laser scanning confocal microscopy of Lenti-X 293T cells co-transfected with the pEGFP-GPI$_{BY55}$ and pmCherry-N1 plasmids. Venn diagram showing the number and percentage of cells for fluorescence negative, EGFP single positive, mCherry single positive and EGFP/mCherry double positive cells.

2 Supplementary Tables

Supplementary Table 1

The membrane targeting signal peptide sequence of given membrane protein

name	N-Signal	GPI/TMD	score	alterations
DAF	1-34th	353th	0.9414	
BY55	1-24th	159th	0.4839	
CEAM7	1-35th	242th	0.5096	
ITAV	1-30th	993-1016th	0.9774	C terminal 4aa removed and 2aa mutated
ITA5	1-41th	996-1021th	0.9481	C-terminal 1aa mutation
ITB3	1-26th	719-741th	0.4499	C terminal 19aa removed

Supplementary Table 2

Amino acid sequences of the six sorting tags

-TST[a]-EGFP[b]-	WSHPQFEK-GGGSGGGSGGSA-WSHPQFEK-PVATMVSKGEELFTGVVPILVELDGDVNGHKFSVSGEGEGDATYGKLTLKFICTTGKLPVPWPTLVTTLTYGVQCFSRYPDHMKQHDFFKSAMPEGYVQERTIFFKDDGNYKTRAEVKFEGDTLVNRIELKGIDFKEDGNILGHKLEYNYNSHNVYIMADKQKNGIKVNFKIRHNIEDGSVQLADHYQQNTPIGDGPVLLPDNHYLSTQSALSKDPNEKRDHMVLLEFVTAAGITLGMDELYKSGRTQI-
DAF Nsig-TST-EGFP-GPI[c]	MTVARPSVPAALPLLGELPRLLLLVLLCLPAVWG-*TST-EGFP*[e]-SRTTKHFHETTPNKGSGTTSGTTRLLSGHTCFTLTGLLGTLVTMGLLT
BY55 Nsig-TST-EGFP-GPI	MLLEPGRGCCALAILLAIVDIQSGG-*TST-EGFP*[e]-RQHLEFSHNEGTLSSGFLQEKVWVM LVTSLVALQAL
CEAM7 Nsig-TST-EGFP-GPI	MGSPSACPYRVCIPWQGLLLTASLLTFWNLPNSAQT-*TST-EGFP*[e]-SRSDPVTLNVRYESVQASSPDLSAGTAVSIMIGVLAGMALI
ITB3 Nsig-TST-EGFP-TM[d]	MRARPRPRPLWATVLALGALAGVGVGGP-*TST-EGFP*[e]-ILYVVEEPECPKGPDILVVLLSVMGAILLIGLAALLIWKLLITIHARKEFAKFEEERARAKADTAN
ITAV Nsig-TST-EGFP-TM	MAFPPRRRLRLGPRGLPLLLSGLLLPLCRAFN-*TST-EGFP*[e]-WGIQPAPMPVPVWVIILAVLAGLLLLAVLVFVMYRMGFFKAVHPPQEEQEREQLQPHENGE
ITA5 Nsig-TST-EGFP-TM	MGSRTPESPLHAVQLRWGPRRRPPLLPLLLLLLPPPPRVGGF-*TST-EGFP*[e]-AVQWTKAEGSYGVPLWIIILAILFGLLLLGLLIYILYKLGFFKASLPYGTAMEKAQLKPPATSDA

a. Twin-Strep-Tag (TST)
b. Enhanced Green Fluorescent Protein (EGFP)
c. Glycosylphosphatidylinositol (GPI)
d. Transmembrane domain (TMD)
e. The sequence of Twin-Strep-Tag and Enhanced Green Fluorescent Protein.

Supplementary Table 3

Primers for constructing the expression plasmids of six sorting tags

Name	Sequence	Note
DAF-Nsig-AgeIF<br>DAF-Nsig-TST-R	AGATCCGCTAGCGCT***ACCGGT***[a]acc**atg**[b]accgtg gctagacccagcg<br>gcctcctcctttctcgaactgagggtggctccaggcgctTCCCCA CACAGCAGGCAGGC	PCR for pGEFP-GPI$_{DAF}$
BY55-Nsig-AgeIF<br>BY55-Nsig-TST-R	AGATCCGCTAGCGCT***ACCGGT***acc**atg**ctgctgga gcctggcagag<br>gcctcctcctttctcgaactgagggtggctccaggcgctGCCTCC GCTCTGGATGTCCAC	PCR for pGEFP-GPI$_{BY55}$
CEAM7-Nsig-AgeIF<br>CEAM7-Nsig-TST-R	AGATCCGCTAGCGCT***ACCGGT***acc**atg**ggcagcc ctagcgcctgccct<br>gcctcctcctttctcgaactgagggtggctccaggcgctGGTCTG GGCGCTGTTGGGCAGG	PCR for pGEFP-GPI$_{CEAM7}$
ITAV-Nsig-AgeIF<br>ITAV-Nsig-TST-R	AGATCCGCTAGCGCT***ACCGGT***acc**atg**gccttccc tcctagacggaga<br>gcctcctcctttctcgaactgagggtggctccaggcgctGTTGA AGGCTCTGCACAGAGGCA	PCR for pGEFP-GPI$_{ITAV}$
ITA5-Nsig-AgeIF<br>ITA5-Nsig-TST-R	AGATCCGCTAGCGCT***ACCGGT***acc**atg**ggcagcc ggacacccgag<br>gcctcctcctttctcgaactgagggtggctccaggcgctGAATC CTCCCACTCTGGGAGGT	PCR for pGEFP-GPI$_{ITA5}$
ITB3-Nsig-AgeIF<br>ITB3-Nsig-TST-R	AGATCCGCTAGCGCT***ACCGGT***acc**atg**agagcta gacctagacctaga<br>gcctcctcctttctcgaactgagggtggctccaggcgctAGGTC CGCCCACTCCCACTCCA	PCR for pGEFP-GPI$_{ITB3}$
EGFP-C2-F<br>EGFP-C2-R	CCGGTCGCCACCATGGTGAG<br>GATCTGAGTCCGGCCGGACTTG	PCR for EGFP
TST-54F<br>TST-59R	TCAGTTCGAGAAAGGAGGAGGCAGCGGCG GCGGAAGCGGAGGCTCCGCTTGGTC<br>CTCACCATGGTGGCGACCGGCTTTTCGAAC TGTGGGTGGGACCAAGCGGAGCCTCCGCT	PCR for TST

a. Endonuclease restriction site recognition sequence of AgeI.
b. Protein translation sequence start codon.

Supplementary Table 4

Primers for constructing CEBPB and CTCF overexpression plasmids and for RT-qPCR analysis.

Name	Sequence	Note
pcDNA3.1-Neo-Up-R<br>pcDNA3.1-Neo-dwn-F	AAACGATCCTCATCCTGTCT<br>GCGGGACTCTGGGGTTCGC	PCR for linearized pcDNA3.1 vector
BY55-Nsig-NeomF<br>BY55-GPI-NeomR	AGACAGGATGAGGATCGTTTACCA TGCTGCTGGAGCCTGGCAGAG<br>GCGAACCCCAGAGTCCCGCtcaCAG GGCCTGGAGAGCCACCA	PCR for Nsig-TST-EGFP-GPI_{BY55}
CEBPB-CDS-F<br>CEBPB-CDS-R	gctAAGCTTatgcaacgcctggtggcct<br>agTCTAGAgcagtggccggaggaggc	PCR for CEBPB CDS region
CTCF-CDS-F<br>CTCF-CDS-R	gctAAGCTTatggaaggtgatgcagtcgaag<br>agTCTAGAccggtccatcatgctgagga	PCR for CTCF CDS region
CEBPB-140f<br>CEBPB-140r	CCCTCGCAGGTCAAGAGCAA<br>TTGTGCTGCGTCTCCAGGTT	RT-qPCR for CEBPB
CTCF-77f<br>CTCF-77r	ATGTGCGATTACGCCAGTGTA<br>TGAAACGGACGCTCTCCAGTA	RT-qPCR for CTCF
EGFP-127f<br>EGFP-127r	ACGACGGCAACTACAAGACC<br>TTGTACTCCAGCTTGTGCCC	RT-qPCR for EGFP
actin151RT-f<br>actin151RT-r	CAGCCATGTACGTTGCTATCCAGG<br>AGGTCCAGACGCAGGATGGCATG	RT-qPCR for β-actin

Supplementary Table 5

Primers for constructing ABL-shRNA1 and ABL-shRNA2 expression plasmid and for RT-qPCR analysis.

Name	Sequence	Note
BY55-Nsig-BamF<br>BY55-GPI-KpnR	AGGGGGATCCACCGGAGCTTACCATGCTGCT GGAGCCTGGCAGAG<br>CAGAGGTACCTCACAGGGCCTGGAGAGCCAC CA	PCR for Nsig-TST-EGFP-GPI_{BY55}
ABL-sh-F1:<br>ABL-sh-R1:	CCGGGCCGAGTTGGTTCATCATCATCTCGAG ATGATGATGAACCAACTCGGCTTTTTG<br>AATTCAAAAAGCCGAGTTGGTTCATCATCAT CTCGAGATGATGATGAACCAACTCGGC	ABL-shRNA1 sequence
ABL-sh-F2:<br>ABL-sh-R2:	CCGGGCAGTCATGAAAGAGATCAAACTCGA GTTTGATCTCTTTCATGACTGCTTTTTG<br>AATTCAAAAAGCAGTCATGAAAGAGATCAA ACTCGAGTTTGATCTCTTTCATGACTGC	ABL-shRNA2 sequence
ABL-130f<br>ABL-130r	ctgagcaggttgatgacagg<br>ctccaaatgcccagacgtcg	RT-qPCR for ABL

Supplementary Table 6

Primers for constructing genome editing plasmids targeting rs1388941 locus and for getPCR analysis.

Name	Sequence	Note
CMVEnhPro-px330-F	GCAGGCATGCTGGGGAGCGGCCTTAT<br>TAATAGTAATCAATTACGGGGTC	PCR for Nsig-TST-EGFP-GPI$_{BY55}$
SV40polyA-px330-R	TCACTAGGGGTTCCTGCGGCCACGCG<br>TTAAGATACATTGATGAGT	
1388941T1-F<br>1388941T1-R	caccGGAGAGAAGAGGGAGAGGAA<br>aaacTTCCTCTCCCTCTTCTCTCC	sgRNA for rs1388941
1388941T1-A-fo4	CCAGGAGAGAAGAGGGAGAGGAAA	getPCR for allele A
1388941T1-G-fo4	CAGGAGAGAAGAGGGAGAGGAAG	getPCR for allele G
1388941T1-r	GATAAAAGGCGACCTGTGGCCC	getPCR for allele A or G
1388941Ctrl-97F<br>1388941Ctrl-97R	TTGGCTGCCCTCCCCGCCGA<br>CAGGGCAGCAGAGACCGCGAC	getPCR for control
HOXB13T6-F<br>HOXB13T6-R	CACCgtgccttatggttactttgg<br>AAACccaaagtaaccataaggcac	sgRNA for HOXB13
HOXB13T6-Fo4-f<br>HOXB13T6-r	tcccgtgccttatggttactttgga<br>CAGTGGGGCGGCTGGGGTA	getPCR for HOXB13
HOXB13-f<br>HOXB13-r	GCGACATGACTCCCTGTTGCCTGTG<br>GACCTGGTGGGTTCTGTTCTCCCTG	getPCR for control
